# *In Vitro* Antimalarial Activity of Inhibitors of the Human GTPase Rac1

**DOI:** 10.1128/AAC.01498-21

**Published:** 2022-01-18

**Authors:** Silvia Parapini, Silvio Paone, Emanuela Erba, Loredana Cavicchini, Manoochehr Pourshaban, Francesco Celani, Alessandro Contini, Sarah D’Alessandro, Anna Olivieri

**Affiliations:** a Dipartimento di Scienze Biomediche per la Salute, Università degli Studi di Milano, Milan, Italy; b Dipartimento di Sanità Pubblica e Malattie Infettive, Sapienza Università di Roma, Rome, Italy; c Dipartimento di Malattie Infettive, Istituto Superiore di Sanità, Rome, Italy; d Dipartimento di Scienze Farmaceutiche, Università degli Studi di Milano, Milan, Italy; e Dipartimento di Scienze Biomediche, Chirurgiche e Odontoiatriche, Università degli Studi di Milano, Milan, Italy; f Dipartimento di Scienze Farmacologiche e Biomolecolari, Università degli Studi di Milano, Milan, Italy

**Keywords:** *Plasmodium*, Rac1, antimalarial drug

## Abstract

Malaria accounts for millions of cases and thousands of deaths every year. In the absence of an effective vaccine, drugs are still the most important tool in the fight against the disease. *Plasmodium* parasites developed resistance to all classes of known antimalarial drugs. Thus, the search for antimalarial drugs with novel mechanisms of action is compelling. The human GTPase Rac1 plays a role in parasite invasion of the host cell in many intracellular pathogens. Also, in Plasmodium falciparum, the involvement of Rac1 during both the invasion process and parasite intracellular development was suggested. The aim of this work is to test a panel of Rac1 inhibitors as potential antimalarial drugs. Fourteen commercially available or newly synthesized inhibitors of Rac1 were tested for antimalarial activity. Among these, EHop-016 was the most effective against P. falciparum *in vitro*, with nanomolar 50% inhibitory concentrations (IC_50_s) (138.8 ± 16.0 nM on the chloroquine-sensitive D10 strain and 321.5 ± 28.5 nM on the chloroquine-resistant W2 strain) and a selectivity index of 37.8. EHop-016 did not inhibit parasite invasion of red blood cells but affected parasite growth inside them. Among the tested Rac1 inhibitors, EHop-016 showed promising activity that raises attention to this class of molecules as potential antimalarials and deserves further investigation.

## INTRODUCTION

Malaria is a vector-borne parasitic disease, which accounts for more than 200 million cases in tropical and subtropical regions. In 2019, an estimated 409,000 deaths occurred, 67% of which were in children under 5 years of age (1). The etiological agent is a protozoan parasite belonging to the genus *Plasmodium*. Among the five species infecting humans, Plasmodium falciparum is the most lethal.

Malaria infection starts when an infected mosquito injects infective stages called sporozoites into the circulating blood. After a single replicative cycle in hepatocytes, *Plasmodium* parasites invade erythrocytes and develop inside red blood cells (RBCs) through two developmental stages, named trophozoites and schizonts. When schizonts are mature, the RBC membrane ruptures, and daughter merozoites are released in the bloodstream, ready to infect new RBCs. The synchronous rupture of infected erythrocytes causes the release of parasite products, responsible for fever and other malaria symptoms. Some of the parasites inside RBCs develop into sexual forms called gametocytes, which can start replication in the mosquito vector.

The only registered vaccine for malaria (RTS,S/AS01) has relatively low efficacy and is not recommended for routine use (2), leaving antimalarial drugs as one of the key tools for malaria control. Unfortunately, *Plasmodium* has developed resistance to all classes of known antimalarial compounds (3–6). Thus, the search for new classes of antimalarials is a relevant field of research.

The human protein Rac1 (Ras-related C3 botulinum toxin substrate 1) is a GTPase involved in several biological processes requiring actin cytoskeleton regulation (7). Interestingly, Rac1 was shown to play an important role in infection by many intracellular pathogens (8–15), including Toxoplasma gondii, which belongs to the same phylum as *Plasmodium* parasites (16). Recently, we investigated the possible involvement of Rac1 also in *Plasmodium* infection of RBCs (17). We showed that Rac1 is recruited in proximity to the site of parasite entry during the invasion process and that it subsequently localizes to the parasitophorous vacuole membrane, the membrane surrounding the parasite during its whole intraerythrocytic life. By using two specific Rac1-inhibitory compounds, EHT1864 and 1A116, we demonstrated that the GTPase plays a role both in the invasion process and in parasite intracellular development (17).

Rac1 acts as a switch by cycling between an active (GTP-bound) and an inactive (GDP-bound) conformation in response to chemical or physical stimuli. The protein is activated by a class of regulatory proteins called guanine nucleotide exchange factors (GEFs), which promote the exchange of GDP for GTP. Rac1 GEFs are of two different types: the so-called “canonical” GEFs, belonging to the Dbl family, and the “atypical” GEFs, belonging to the DOCK family (18). On the other side, the GTPase-activating proteins (GAPs) negatively regulate Rho-mediated signals by binding to activated forms of Rho GTPases and stimulating GTP hydrolysis.

Because of its role in the spread of many tumors, Rac1 has been widely studied, and several Rac1 inhibitors have been developed and are commercially available. Rac1 inhibitors may target different functions of the protein, such as its interaction with GEFs and GAPs, its nucleotide exchange activity, protein localization, its stability, and its downstream effectors (19).

Among the inhibitors targeting the interaction between Rac1 and GEFs (Table 1), NSC23766 binds to the region containing the Trp56 residue, which is critical for the interaction with the GEF T-lymphoma invasion and metastasis-inducing protein 1 (Tiam1) and triple functional domain protein (Trio) (20). Based on NSC23766, several other Rac1 inhibitors binding to the same region of the protein were designed, such as EHop-016 and Aza-1 (21, 22). In particular, EHop-016 blocks Rac1 interaction with the GEF Vav guanine nucleotide exchange factor 2 (Vav2) (21).

**TABLE 1:** Rac1 chemical inhibitors^*a*^

Rac1 inhibitor (reference)	Specificity(ies)	IC_50_ (μM)	Activity in cell type
NSC23766 (20)	Tiam1, Trio/Rac1	20–50	Rac1 activity in PDGF-stimulated NIH 3T3 fibroblasts
EHop-016 (21)	Vav2/Rac1	1.1	Rac1 activity in MDA-MB-435 melanoma cells
Aza-1 (22)	GEFs	5–20	Rac1 activity in 22Rv1 human prostate cancer cells
Rac1 inhibitor II (24)	Tiam1/Rac1	12.2	Rac1 activity in human smooth muscle cells
Compound 3 (25)	GEFs	16.4	Rac1 activity in human smooth muscle cells
Compound 4 (25)	Tiam1, Vav2/Rac1	8.7	Rac1 activity in human smooth muscle cells
ZINC69391 (26)	Tiam1, Dock180/Rac1	61	F3II mammary carcinoma cell proliferation
1A116 (26)	Tiam1, Dock180, P-Rex1/Rac1	4	F3II mammary carcinoma cell proliferation
ITX3 (27)	Trio/Rac1	50–100	Actin stress fiber formation in REF-52 rat embryo fibroblasts
W56 (28)	Tiam1/Trio	100–150	Rac-GEF interaction *in vitro*
EHT1864 (29)	Nucleotide binding	10–50	Rac1 activity in U87MG glioma cells
MLS000532223 (30)	Nucleotide binding	∼10	Rac1 activity in Swiss 3T3 embryonic mouse fibroblasts
ML141 (31)	Nucleotide binding	∼10	Rac1 activity in EGF-stimulated NIH 3T3 fibroblasts

a For each Rac1 inhibitor, the inhibitory specificities and the IC_50_ values on different cell types reported in the literature are summarized. PDGF, platelet-derived growth factor; EGF, epidermal growth factor.

Other chemical inhibitors affecting Rac1 binding to GEFs were developed by virtual screening of molecules from the ZINC database and then tested *in vitro* for their inhibitory activity (23). Among them, Rac1 inhibitor II inhibits binding to the GEF Tiam1 (24), whereas compounds 3 and 4 (see Materials and Methods) inhibit binding to the GEFs Trio, Tiam1, and Vav2 (25). Also, ZINC69391 and its derivative 1A116 inhibit Rac1 binding to the GEF Tiam1 but also to the dedicator of cytokinesis (Dock180), an atypical GEF from the DOCK family. 1A116 also inhibits Rac1 binding to the GEF phosphatidylinositol-3,4,5-trisphosphate-dependent Rac exchange factor 1 (P-Rex1) (26). ITX3 specifically inhibits the bond to the GEF Trio although with rather low efficiency (27). W56 is a peptide derived from the Rac1 GEF-binding region that reduces Rac1-GEF interactions by competing with the endogenous Rac1 protein, with specificity against the GEFs Tiam1 and Trio (28) but with low efficacy (Table 1).

Preventing Rac1 binding to nucleotides, rather than its activation by GEFs, is an alternative inhibitory strategy. EHT1864 displaces guanine nucleotides from the catalytic site, keeping Rac1 in an inactive, inert state (29); MLS000532223 and ML141 also inhibit nucleotide binding (30, 31).

The aim of the present work was to test a series of Rac1 inhibitors for potential antimalarial activity.

## RESULTS

### Antimalarial activity of Rac1 inhibitors.

**FIG 1:**
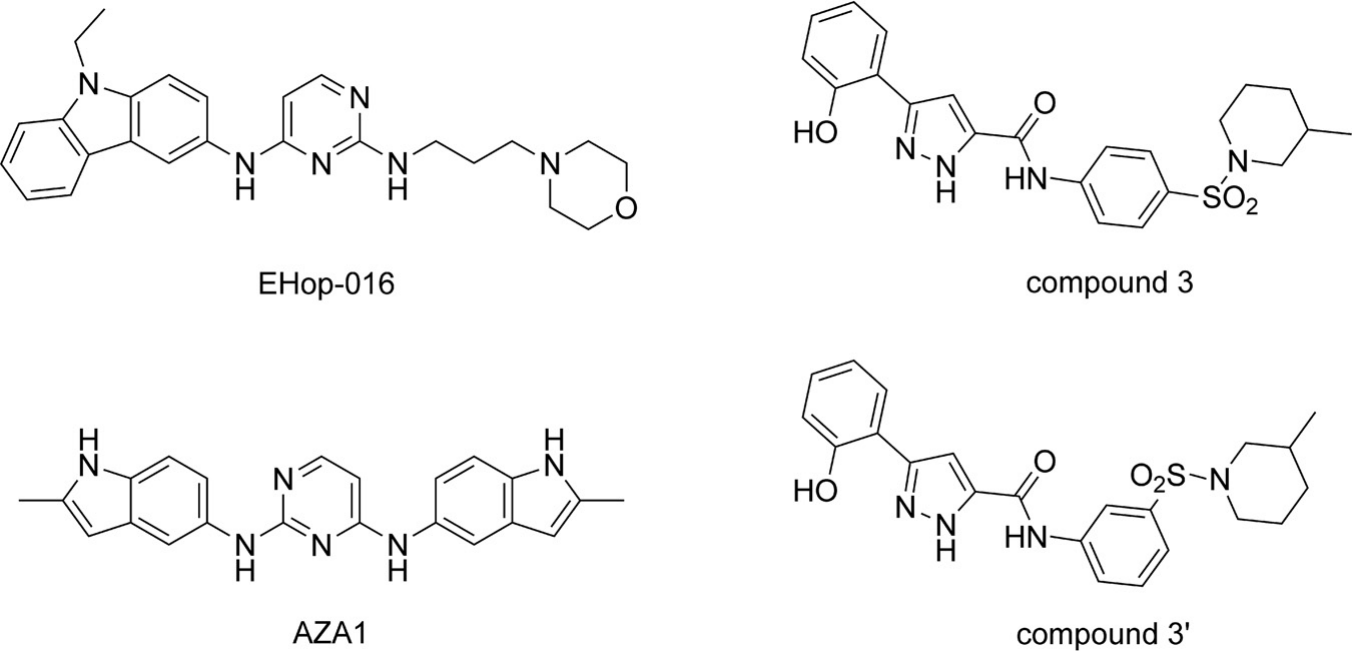
The most effective Rac1 inhibitors (EHop-016, Aza-1, and compound 3) on P. falciparum and comparison between the structures of compound 3 and compound 3′.

Three different inhibitors of Rac1 guanosine nucleotide exchange activity and 11 inhibitors of the interaction between Rac1 and its GEFs were tested against asexual parasites of the chloroquine (CQ)-sensitive D10 and CQ-resistant W2 strains. Results are reported in Table 2. Three inhibitors had a 50% inhibitory concentration (IC_50_) <1 μM, and two were inactive (IC_50_ > 50 μM), whereas the remaining inhibitors showed intermediate IC_50_s on both strains. Even if structurally unrelated, the three most active compounds (EHop-016, Aza-1, and compound 3) (Fig. 1) were inhibitors of Rac1 interaction with GEFs. Interestingly, compounds 3 and 4 were previously reported to be similarly potent when tested on smooth muscle cells as Rac1 inhibitors (25). Conversely, when evaluated on P. falciparum strains, they behaved differently, with the former compound being much more effective (11- and 48-fold on the D10 and W2 strains, respectively). Compound 3′, which is an isomer of compound 3 (Fig. 1) never reported previously, showed intermediate efficacy between compounds 3 and 4, with IC_50_s of 2.76 ± 0.12 and 1.24 ± 0.13 μM on the D10 and W2 strains, respectively. Since compounds 3 and 3′ have a stereocenter at C-3 on the piperidine ring, we also prepared and evaluated the pure (*S*)-enantiomer for both compounds. However, activity data on the D10 and W2 strains were superimposable with those obtained by the racemate (see Table S1 in the supplemental material).

**TABLE 2:** Activity of Rac1 inhibitors against P. falciparum asexual parasites and gametocytes^*a*^

Inhibitor	Mean IC_50_ (μM) ± SD			
	D10	W2	3D7 immature gametocytes	3D7 mature gametocytes
ML141	10.70 ± 0.29	17.26 ± 1.84		
EHT1864^*b*^	3.88 ± 0.74	7.75 ± 1.82	>50.00	32.59 ± 0.92
1A116^*b*^	3.21 ± 1.17	6.89 ± 1.41	78.13 ± 17.68	56.64 ± 21.34
NSC23766	5.02 ± 1.47	4.05 ± 0.50	15.33 ± 3.70	30.83 ± 3.09
Rac1 inhibitor II	11.89 ± 7.37	37.47 ± 8.67		
EHop-016	0.14 ± 0.02	0.32 ± 0.03	0.74 ± 0.20	4.65 ± 0.52
ZINC69391	17.50 ± 4.64	12.56 ± 5.25		
W56	>50.00	>50.00		
Aza-1	0.25 ± 0.03	0.76 ± 0.27	1.25 ± 0.48	7.23 ± 2.12
MLS000532223	6.89 ± 0.73	9.51 ± 1.66		
ITX3	>50.00	>50.00		
Compound 3	0.66 ± 0.05	0.52 ± 0.02	2.26 ± 0.22	21.85 ± 2.71
Compound 4	7.14 ± 0.41	25.42 ± 3.98		
Compound 3′	2.76 ± 0.12	1.24 ± 0.13	2.13 ± 0.43	26.58 ± 3.70
Methylene blue			0.03 ± 0.01	0.12 ± 0.03
Chloroquine	0.02 ± 0.003	0.42 ± 0.08		

a Data are the means ± standard deviations from at least 3 independent experiments in duplicate.

b Data for EHT1864 and 1A116 were reported previously (17).

We previously showed that Rac1 is the only Rac GTPase present in mature erythrocytes (17). ML141 and Aza-1 also inhibit the closely related Rho GTPase CDC42 (22, 31). To exclude the possible involvement of this GTPase in *Plasmodium* infection, we tested two inhibitors specific for CDC42 (ZCL278 and MLS57315) on P. falciparum cultures (30, 32). These compounds were not active against malaria parasites (Table S2), at neither sexual nor asexual stages, excluding that the antimalarial activity of the Rac1 inhibitors is due to off-target effects on CDC42.

The compounds with IC_50_s <5 μM (on at least one strain) were also tested for activity against immature and mature gametocytes. All the molecules had lower activity on gametocytes than on asexual parasites, in particular on the mature stages. This was not surprising because mature gametocytes are usually less sensitive to drugs (33, 34).

EHop-016 was the most active against all the malaria parasite stages.

CQ (35) and methylene blue (MB) (36) were used as positive controls for asexual stages and gametocytes, respectively. EHT1864 and 1A116 had previously been tested on asexual parasites, and the resulting IC_50_s are reported in Table 2, for comparison (17).

### Rac1 inhibitors show moderately good selectivity indices.

Inhibitors with IC_50_s <5 μM (on at least one strain) were tested for cytotoxicity against HMEC-1 human microvascular endothelial cells (Table 3). The highest selectivity indices (SIs) were obtained with EHop-016, Aza-1, and 1A116.

**TABLE 3:** Cytotoxicity against HMEC-1 cells and selectivity indices^*a*^

Rac1 inhibitor	Mean HMEC-1 IC_50_ (μM) ± SD	SI
EHop-016	5.22 ± 1.07	37.8
EHT1864	48.47 ± 1.23	12.5
Aza-1	12.32 ± 6.11	48.9
1A116	131.50 ± 37.26	41.1
NSC23766	153.77 ± 38.51	30.6
Compound 3′	16.00 ± 0.44	5.8
Compound 3	10.59 ± 1.19	16.1

a SI, selectivity index, calculated as the ratio between the IC_50_ against HMEC-1 cells and the IC_50_ against D10 asexual parasites.

### Effect of EHop-016 on P. falciparum invasion and intraerythrocytic growth.

The Rac1 inhibitors previously used to investigate the role of Rac1 in malaria infection, EHT1864 and 1A116, were selected for their ability to inhibit Rac1 activity regardless of its molecular environment (17). EHT1864 inhibits Rac1 by directly binding to its catalytic site (29), and 1A116 inhibits Rac1 interactions with both “canonical” and “atypical” GEFs (26). Proteomic analysis of human erythrocytes did not identify any canonical GEFs, while some atypical ones were identified (37, 38), leading to the idea that Rac1 in erythrocytes may be activated by an atypical GEF.

For this reason, we found it surprising that the most effective among the Rac1 inhibitors was EHop-016, which specifically inhibits Rac1 interaction with the canonical GEF Vav2 and was not supposed to inhibit Rac1 in erythrocytes. We thus decided to characterize in more detail its effects on specific stages of the parasite life cycle.

**FIG 2:**
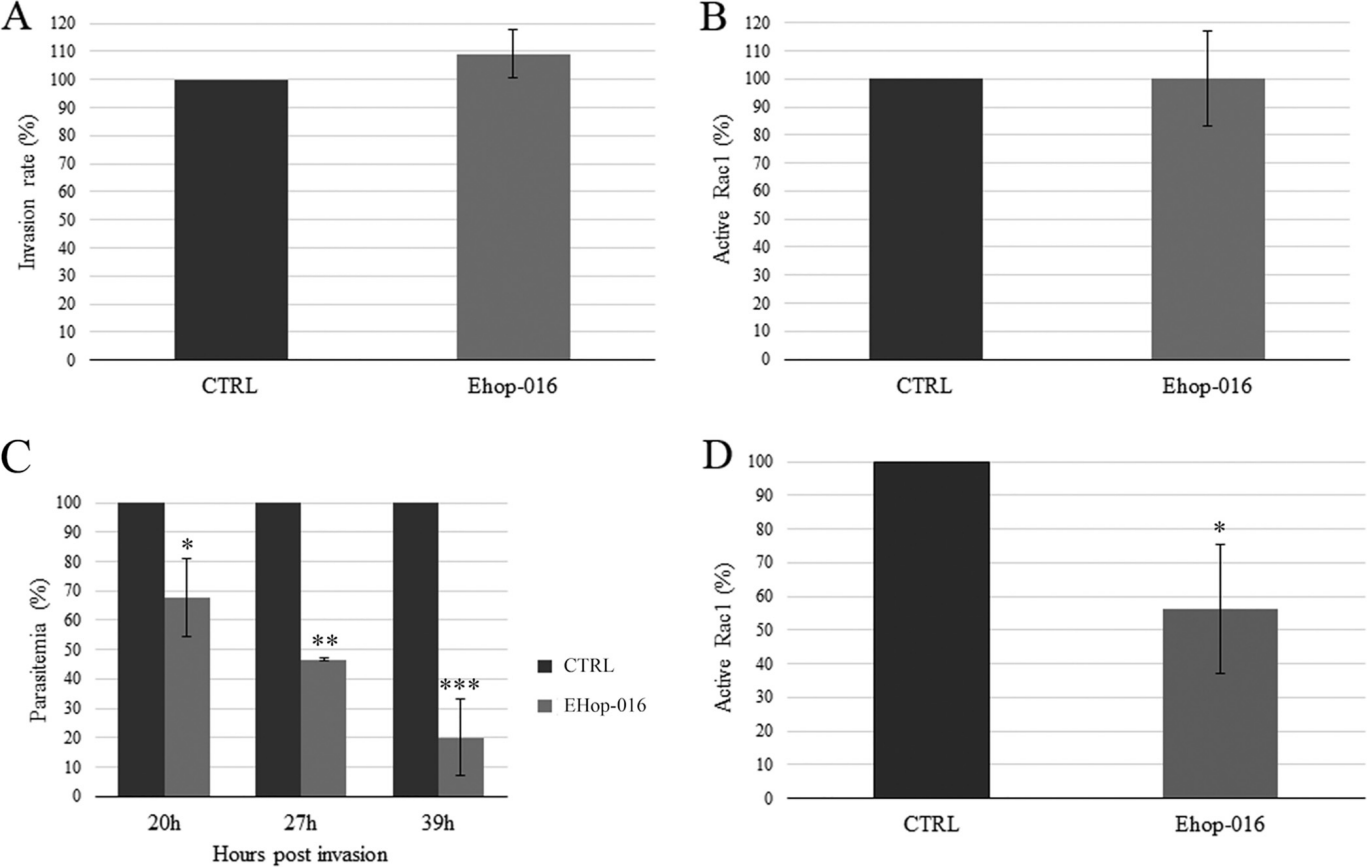
Effect of EHop-016 on P. falciparum invasion and intraerythrocytic growth. (A) P. falciparum invasion rates in the presence of 2.5 μM EHop-016 compared to the untreated control. (B) Levels of active Rac1 in uninfected erythrocytes treated with 2.5 μM EHop-016 compared to the untreated control. (C) Parasitemia of infected erythrocytes treated with 1.4 μM EHop-016 expressed as a percentage of the untreated control, at 20, 27, and 39 hpi. *, *P* < 0.02; **, *P* < 0.001; ***, *P* < 0.01 (by Student's *t* test). (D) Levels of active Rac1 in 1.4 μM EHop-016-treated parasites at 20 hpi, expressed as a percentage of the untreated control. *, *P* < 0.001 (by Student's *t* test).

We performed invasion assays using EHop-016 at a dose of 2.5 μM (about 20 times the IC_50_ assessed for asexual parasites of the D10 strain). As expected, this inhibitor did not reduce the RBC invasion efficiency (Fig. 2A). We then investigated the Rac1 activation state in erythrocytes treated with 2.5 μM EHop-016 and in the untreated control by G-LISA, a commercial kit specifically designed to measure the amount of active Rac1. This showed that EHop-016 does not inhibit Rac1 in noninfected erythrocytes (Fig. 2B), supporting the hypothesis that Rac1 may be activated by an atypical GEF in erythrocytes.

We then investigated the effect of EHop-016 on parasite intraerythrocytic growth and survival. Synchronous parasites were cultured in the presence of the inhibitor at a 1.4 μM concentration (about 10 times the IC_50_ assessed for D10 strain asexual parasites, below the concentration used in invasion assays). Blood smears were taken at 20, 27, and 39 h post-invasion (hpi), when parasites were almost mature but none of them had yet released daughter merozoites. Parasitemia was assessed by counting parasites on Giemsa-stained smears. EHop-016 caused a significant, time-dependent reduction in parasitemia, resulting in a dramatic drop in the final number of vital parasites at 39 hpi, compared to the control (Fig. 2C). Moreover, parasites treated with EHop-016 showed a complete block of the developmental process. None of the treated parasites developed into the trophozoite stage, as indicated by the absence of hemozoin, the product of hemoglobin catabolism, which is a distinctive feature of the trophozoite stage. At 39 hpi, dotty forms are visible inside erythrocytes, presumably representing parasite residues after death by shrinkage (Fig. S3A). These results showed that EHop-016 is highly efficient and selective in blocking parasite intraerythrocytic development.

We thus decided to investigate the Rac1 activation state in infected erythrocytes treated with EHop-016 and in the untreated control. To do this, we applied a methodology that allows us to specifically measure Rac1 activation levels on the parasitophorous vacuole. Freshly invaded erythrocytes were thus exposed to 1.4 μM EHop-016 treatment for 20 h and then smeared and analyzed by an immunofluorescence assay (IFA) with an antibody that specifically recognizes Rac1 in its active form (anti-Rac1/GTP). The resulting fluorescence signal was measured both in the EHop-016-treated sample and in the untreated control.

When comparing the fluorescence intensities of parasitophorous vacuoles, the signal was significantly lower in the EHop-016-treated sample than in the untreated control (Fig. S3B), indicating that, unexpectedly, EHop-016 can efficiently inhibit Rac1 on the parasitophorous vacuole. This result is suggestive that in this location, the GTPase may be activated by a parasite protein mimicking a human canonical GEF.

### Association of EHop-016 and 1A116 on P. falciparum asexual parasite growth.

**FIG 3:**
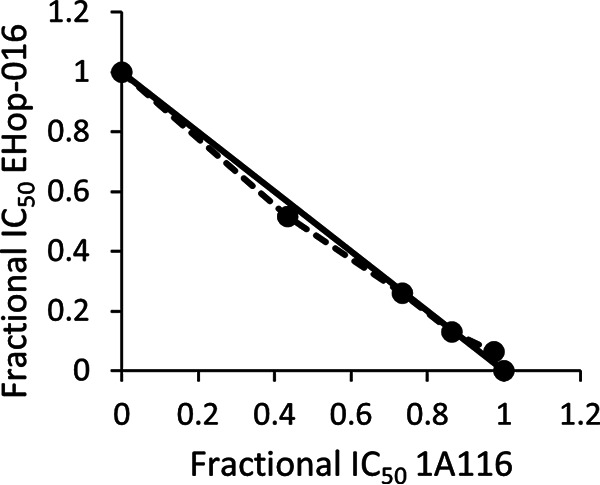
Isobologram analysis of the antimalarial activity of EHop-016 in combination with 1A116. The two compounds showed additive activity. Results are the means from three independent experiments.

Since EHop-016 and 1A116 have different specificities for GEFs, IC_50_s against asexual parasites <5 μM (on at least one strain), and high selectivity indices, they were tested in combination studies using W2 parasites. These experiments indicated that EHop-016 had an additive effect with 1A116 (fractional inhibitory concentration [FIC] of <2), as shown by isobologram analysis (Fig. 3).

## DISCUSSION

A panel of Rac1 inhibitors, including modulators of nucleotide binding and inhibitors of the interaction between Rac1 and its GEFs, was tested for antimalarial activity against asexual and sexual P. falciparum parasites. Twelve compounds, with different chemical structures and different inhibition mechanisms, showed antimalarial activity. Two of them had no effect (ITX3 and W56). This is not surprising since these two compounds also showed low efficacy on different cell types (19). EHop-016, Aza-1, and compound 3 were the most active against all stages of malaria parasites, with IC_50_s below 1 μM on asexual stages.

Two compounds inhibiting the closely related Rho GTPase CDC42 were not active against malaria parasites, thus excluding possible off-target effects on this GTPase.

Interestingly, the structurally related compounds 3 and 4, which were previously reported to be similarly potent when tested on smooth muscle cells, behaved rather differently on P. falciparum cultures, with compound 3 being between 10 and 50 times more potent than compound 4, depending on the evaluated parasite strain. This is surprising since they differ only by a methyl group on the piperidine ring (Fig. 1). The newly synthesized compound 3′, an isomer of compound 3 bearing a *meta* substitution pattern at the sulfonylanilino group, was also shown to be more effective than compound 4 but less effective than compound 3. Given that the *para*-sulfonylanilino group is the preferred scaffold for this class of compounds, the substitution at the piperidine ring seems to play an important role in activity and will be the subject of future structure-activity relationship studies.

EHop-016 was chosen for deeper investigation because its IC_50_ was the lowest on both asexual and sexual stages. We showed that EHop-016 does not inhibit Rac1 in uninfected erythrocytes, but it affects the GTPase very efficiently when it is internalized by the parasite. Consistently, it does not affect erythrocyte invasion but strongly affects parasite intraerythrocytic growth and development. Although we cannot exclude that the phenotypes observed are due to off-target effects on other proteins, in both the human host and the parasite, the correlation of EHop-016 detrimental effects on parasites with its inhibitory activity on Rac1 supports the hypothesis that the GTPase may be necessary for parasite survival.

An interesting result is that EHop-016 was shown to be highly efficient in inhibiting Rac1, even though no canonical GEFs are known to be expressed in mature erythrocytes. This result suggests that when internalized into the parasitophorous vacuole, Rac1 may be activated by a parasite protein mimicking a human canonical GEF. This mechanism has previously been reported for several intracellular pathogens, producing factors that mimic host components to modulate Rac1 activity (39). As EHop-016 was the most effective compound and given its selectivity against the GEF Vav2 (21), it is possible that *Plasmodium* parasites activate Rac1 by a GEF-like protein possibly similar to Vav2. Identifying such a parasite protein would be invaluable not only for the basic knowledge of *Plasmodium* biology but also for targeted and specific drug design.

As EHop-016 has been extensively tested as an antitumor agent, data on toxicity and pharmacokinetics in animal models are available (40, 41). EHop-016 does not affect the viability of human mammary epithelial cells at concentrations lower than 5 μM. When administered orally to mice at 25 mg/kg of body weight three times a week for 55 days, it did not cause any significant changes in weight or behavior. The EHop-016 maximum plasma concentration, after the administration of 10, 20, and 40 mg/kg, ranged between 163 and 834 ng/mL, with an elimination half-life ranging from 3.8 to 5.7 h (40, 41). Our data on human endothelial cells confirm the low *in vitro* toxicity of EHop-016. Moreover, thinking of EHop-016 as a future possible antimalarial drug, one of the strategies to reduce toxicity but also drug resistance insurgence is to associate it with another compound with different properties, such as different mechanisms of actions and pharmacokinetics. This has been largely adopted in antimalarial treatment, as testified by the case of artemisinins (42). We tested the association of EHop-016 with 1A116, and the two molecules had an additive effect. This result is compatible with the fact that the two compounds inhibit different stages of parasite development. This additivity may allow the use of lower doses of compounds in a potential future therapeutic regimen.

Targeting host molecules that pathogens exploit to enter or develop inside the host cell is a novel strategy to limit the insurgence of drug resistance. Host-targeted compounds are less likely to generate drug resistance than conventional antimicrobial therapies since the pathogen needs to redirect its entire infection strategy to compensate for a missing interaction with a host molecule. In fact, the majority of mutations that confer resistance to known antimalarial drugs are point mutations in parasite genes encoding membrane molecular pumps and channels. These mutations increase the efflux of antimalarial compounds from the parasite cytoplasm, thus decreasing their efficacy (43). Such an evolutionary strategy cannot be applied to drugs targeting host molecules. Host-targeted molecules have already been proven to be effective against different intracellular pathogens such as Mycobacterium tuberculosis (44), Helicobacter pylori (45), hepatitis C virus (46), and dengue virus (47), and two are already in clinical use for hepatitis C and AIDS treatment (48–50). For malaria, an example is conoidin A, an irreversible inhibitor of the host peroxidase Prx2 that impairs DNA synthesis and hemozoin production, disrupts nuclear integrity, and prevents the growth of P. falciparum, making it more sensitive to chloroquine (51). The host-targeting approach also has the advantage of being effective against different parasite strains and possibly also against different parasite species (52–55).

Altogether, these data support the importance of human Rac1 as a potential drug target for the development of antimalarial drugs. Although the mechanisms of action of EHop-016 are still to be clarified, its activity against malaria parasites is promising and worth further investigation.

## MATERIALS AND METHODS

### Inhibitors of Rac1 activity.

The structures of all inhibitors are reported in Fig. 1 and Fig. S1 in the supplemental material. Commercially available inhibitors of Rac1 guanosine nucleotide exchange activity or inhibitors of the interaction between Rac1 and its GEFs were purchased from Aobious (ZINC69391), Focus Biomolecules (MLS000532223), EMD Millipore (Aza-1), Tocris (NSC23766), Merck (EHop-016, ML141, and Rac1 inhibitor II), and Enamine (compound 4). Compounds 3 and 3′ were synthesized by adapting protocols reported in the literature (56, 57), as described in Fig. S2 in the supplemental material.

### Parasite cultures.

Chloroquine (CQ)-sensitive (D10, wild-type 3D7, or transgenic 3D7elo1-pfs16-CBG99) and CQ-resistant (W2) strains of P. falciparum were cultured as described previously (58), with slight modifications, as detailed in the supplemental material.

### Drug susceptibility assays on P. falciparum.

The Rac1 inhibitors and the reference drugs (CQ and methylene blue [MB]) were dissolved in either water, complete medium, or dimethyl sulfoxide (DMSO) to a concentration of 5 or 10 mg/mL, depending on compound solubility. Inhibitors were serially diluted in 96-well flat-bottom microplates (starting from 50 μM; the final DMSO concentration was ≤1%, a concentration not toxic to parasites). Asynchronous asexual parasites or gametocytes (immature or mature stages) were added to the plates (1 to 1.5% parasitemia and 1% final hematocrit) and incubated for 72 h at 37°C. Asexual parasite growth was determined spectrophotometrically (optical density at 650 nm [OD_650_]) by measuring the activity of parasite lactate dehydrogenase (pLDH), according to a modified version of the method of Makler et al. (59). Gametocyte viability was measured by a luminescence method described previously (60). Details for the two methods are reported in the supplemental material. From either the OD or ALU (arbitrary luminescence units), the percentages of viability were calculated according to the formula 100 × (treated sample signal − blank signal)/(untreated control signal − blank signal). The blank for the pLDH assay was uninfected RBCs at the same hematocrit as that of parasitized RBCs, and the blank for the luminescence assay was parasites treated with a high dose of methylene blue. Antimalarial activity was determined as the concentration of drugs inducing 50% growth inhibition (IC_50_).

### Cell cytotoxicity assays.

Cytotoxicity was evaluated on human microvascular endothelial cells (HMEC-1) as detailed in the supplemental material. Cell proliferation was evaluated by an MTT [3-(4,5-dimethylthiazol-2-yl)-2,5-diphenyltetrazolium bromide] assay (61) after 72 h at 37°C with 5% CO_2_. Plates were then read on a microplate reader (Synergy 4; BioTek) at a wavelength of 550 nm (reference, 650 nm). Results are expressed as the IC_50_, the dose necessary to inhibit cell growth by 50%. All tests were performed in duplicate at least three times.

The selectivity index (SI) is the ratio between the cytotoxic IC_50_ value against HMEC-1 cells and the parasitic IC_50_ value against the D10 strain and indicates the compound selectivity for the parasite.

### G-LISAs.

Whole blood collected from 7 donors was washed in RPMI 1640 four times to remove plasma, platelets, and leukocytes. The number of erythrocytes was determined by using a cell counting chamber. Erythrocytes were incubated for 2 h at 37°C with or without 2.5 μM EHop-016 and then washed. In each sample, the Rac1 activation state was measured with the G-LISA Rac1 activation assay luminescence-based biochemical kit (Cytoskeleton). Luminescence intensity was assessed with a BioTek Synergy HT plate reader. These experiments were performed in three biological replicates.

### Association experiments.

The effect of 1A116 on the IC_50_ of EHop-016 was determined by potentiation tests as previously described (62). Isobolograms were created by plotting a pair of fractional IC_50_s for each combination of EHop-016 and 1A116. The EHop-016 fractional IC_50_ was calculated by dividing the IC_50_ of EHop-016 combined with 1A116 by the IC_50_ of EHop-016 alone, and these data were plotted on the horizontal axis. The corresponding 1A116 fractional IC_50_s were calculated by dividing each fixed concentration by the IC_50_ of 1A116 alone and plotted on the vertical axis. If the isobologram is close to the diagonal, an additive effect between the two drugs is demonstrated. Curves significantly above or below the diagonal indicate antagonistic or synergic effects, respectively.

### Evaluation of parasite invasion efficiency.

Invasion assays were performed as previously described (17). Invasion rates were calculated by subtracting the number of schizonts at the end of the assay (6 h postinvasion [hpi]) from the number of schizonts in the time zero (*T*_0_) sample, obtaining the number of merozoite-releasing schizonts. The number of new infections was normalized to the number of merozoite-releasing schizonts to obtain the parasitized erythrocyte multiplication rate (PEMR). This value was normalized to the control sample, obtaining the invasion rate value. The results are the means from three independent biological replicates, each performed in triplicate.

### Evaluation of parasite intraerythrocytic growth.

A culture of erythrocytes freshly invaded by P. falciparum parasites was set up as previously described (17). Parasites were exposed to 1.4 μM EHop-016, and an untreated control was set up. Blood smears were taken at 20, 27, and 39 hpi, corresponding to late ring, trophozoite, and schizont stages, and then Giemsa stained and counted by optical microscopy in order to assess parasitemia. Pictures of the blood smears were taken with a Leica ICC50W camera. These experiments were performed in three biological replicates.
