# Supplementary material for: In Vitro Antimalarial Activity of Inhibitors of the Human GTPase Rac1
Source: Antimicrob Agents Chemother. 2022 Jan 18;66(1):e01498-21. doi: 10.1128/AAC.01498-21 (PMC8765435; doi:10.1128/AAC.01498-21)
Supplement: Supplemental file 1 — Supplemental material. Download AAC.01498-21-s0001.pdf, PDF file, 1.1 MB [file aac.01498-21-s0001.pdf]

Supplementary information

In vitro antimalarial activity of inhibitors of the human GTPase Rac1

Silvia Parapini[a], Silvio Paone[b,c], Emanuela Erba[d], Cavicchini Loredana[e]†, Manoochehr Pourshaban[b], Francesco Celani[c], Alessandro Contini[d], Sarah D'Alessandro[f*], Anna Olivieri[c*]

[a] Dipartimento di Scienze Biomediche per la Salute, Università degli Studi di Milano, Milan, Italy;

[b] Dipartimento di Sanità Pubblica e Malattie Infettive, Sapienza Università di Roma, Rome, Italy;

[c] Dipartimento di Malattie Infettive, Istituto Superiore di Sanità, Rome, Italy;

[d] Dipartimento di Scienze Farmaceutiche, Università degli Studi di Milano, Milan, Italy;

[e] Dipartimento di Scienze Biomediche, Chirurgiche e Odontoiatriche, Università degli Studi di Milano, Milan, Italy;

[f] Dipartimento di Scienze Farmacologiche e Biomolecolari, Università degli Studi di Milano, Milan, Italy

*Sarah D'Alessandro and Anna Olivieri contributed equally to this work

Materials and methods

Parasite cultures

The CQ-sensitive (D10) and the CQ-resistant (W2) strains were maintained at 5% hematocrit (HCT) (human type A-positive red blood cells) in RPMI 1640 (EuroClone) medium, supplemented with 1% AlbuMaxII (lipid-rich bovine serum albumin) (Invitrogen), 0.01% hypoxantine (Sigma), 20mM Hepes (EuroClone), 2 mM glutamine (EuroClone). The 3D7

strain (wild type or transgenic 3D7elo1-pfs16-CBG99 strain) was cultured in similar conditions, except for 10% naturally clotted Human O+ serum instead of AlbuMaxII and human type O-positive instead of A-positive RBCs. All the cultures were maintained at 37°C in a standard gas mixture consisting of 1% O_2, 5% CO_2, 94% N_2. Immature (stage II-III) and mature (stage V) gametocytes were obtained as described[1]. Briefly, cultures were diluted to 0.5% parasitemia at 5% HCT, and the medium was changed daily until parasites were stressed by nutrient deprivation. N-acetylglucosamine (NAG) (Sigma-Aldrich) 50 mM was then added to clear residual asexual parasites: Stage II and III gametocytes were obtained 4 days after NAG addition to the culture, while stage IV and V gametocytes were used 11 to 13 days from NAG addition. Gametocyte stages were routinely checked on Giemsa-stained smears.

In vitro P. falciparum drug susceptibility assay

After 72h incubation with drugs, 20 µl of parasite culture from each well of the experiment plate were transferred to a plate containing 100µl of Malstat reagent (0.11% [vol/vol] Triton-100, 115.7mM lithium L-lactate, 30.27 mM Tris, 0.62 mM 3-acetylpyridine adenine dinucleotide [APAD] [Sigma-Aldrich], adjusted to pH 9 with 1 M HCl) and 25 µl of PES/NBT (1.96 mM nitroblue tetrazolium chloride-0.24 mM phenazine ethosulfate) to perform the pLDHassay. The plate was read at a wavelength of 650 nm using a microplate reader, Synergy4 (BioTek), and the results were expressed as the 50% inhibitory concentration (IC_{50}). Uninfected RBC at the same HCT than parasites were used as blank.

Drug treated gametocyte cultures were transferred to 96-well black microplates and D-luciferin (1 mM in citrate buffer 0.1 M, pH 5.5) was added at a 1:1 volume ratio. Luminescence measurements were performed after 10 min with 500 ms integration time. Gametocytes treated with 1 µM methylene blue, which completely kills gametocytes, were used as blank.

Cell cytotoxicity assays

Cytotoxicity was evaluated on Human Microvascular Endothelial Cells (HMEC-1) maintained in MCDB-131 medium supplemented with 10% foetal calf serum (Euroclone), 20mM HEPES and 2 mM L-glutamine. Cells were seeded in 96 well flat bottom tissue culture clusters (1.5×10^4 cells/well), let adhere overnight and treated with serial dilutions of test compounds.

Immuno-fluorescence assay

Synchronous mature schizonts were purified by 60% Percoll gradient and allowed to invade fresh human RBCs for two hours. The culture was then treated with 5% sorbitol to kill all parasites except young stages resulting from the recent invasion[2]. Blood smears were taken at 20 hpi from the 1,4 µM EHop-016 treated culture and untreated control and fixed in 4% paraformaldehyde/0.015% glutaraldehyde for 30 min at room temperature. Cells were then permeabilized with 0,1% Triton-X100 in PBS for 10 min and incubated for 1 h with the following primary antibodies: anti-Rac1/GTP mouse monoclonal antibody (NewEast Bio) 1:100 dilution, anti-Exp1 rabbit polyclonal [3] 1:50 dilution. After washing in PBS, samples were incubated with the secondary antibodies: anti-mouse fluorescein (Invitrogen) 1:200 dilution and anti-rabbit rhodamine (ThermoFisher) 1:200 dilution and with the nuclear marker DAPI (Life Technologies) 500 ng/ml. Samples were washed in PBS and smears were mounted in Vectashield (Vector Laboratories). Negative controls without primary antibodies have been performed, resulting in complete absence of fluorescence signals. Anti-Rac1/GTP fluorescence signal was measured by using the software ImageJ (the parasitophorous vacuole area was delimited based on the anti-Exp1 signal). At least 40 cells were analyzed in each sample.

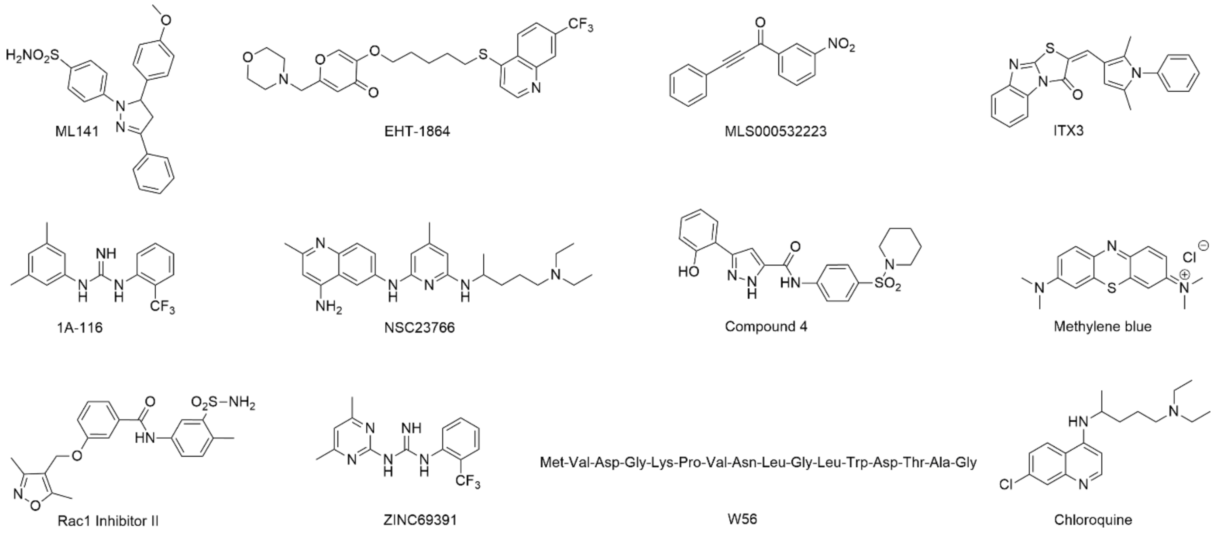

Figure S1. Structure of Rac1 inhibitors and of chloroquine (reference drug).

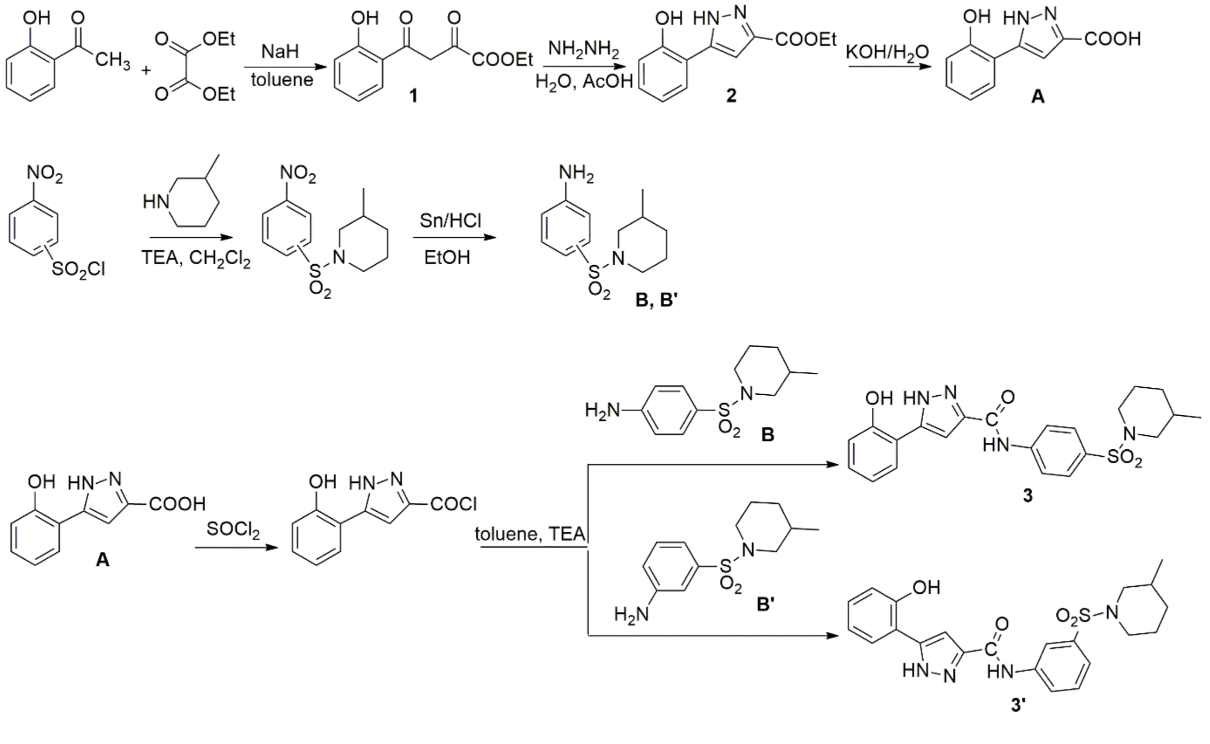

Figure S2. Synthetic scheme for the preparation of compounds 3 and 3'.

Synthesis of compounds 3 and 3'

3-(2-Hydroxyphenyl)-1H-pyrazole-5-carboxylic acid (A, Figure S2) was prepared by adapting protocols reported in the literature.[4, 5] 2-Hydroxyacetophenone (7.3 mmol) was dissolved in

toluene (60 ml) and reacted with NaH (36.5 mmol) under stirring for 10’. Diethyl oxalate (11 mmol) was then added dropwise and the mixture was heated at 50 °C for 1h. The reaction was quenched with 50 ml of water and the solution was cooled in an ice bath, acidified with HCl 2N up to pH = 2 and extracted with AcOEt. The organic layer was anhydrified and evaporated in vacuo, yielding 5.8 mmol of ethyl 4-(2-hydroxyphenyl)-2,4-dioxobutanoate **1** (m.p. 77-78 °C). Compound **1** (5.8 mmol) was dissolved in water (10 ml) and AcOH 10% (7.5 ml) and reacted with hydrazine (18 mmol) under reflux for 1.5 h. The reaction was let to stand overnight and filtered, yielding 4.8 mmol of product **2** (m.p. 119-121 °C). Compound **2** (4.7 mmol) was dissolved in EtOH (5 ml) and water (1 ml) and reacted with KOH (10.7 mmol) under reflux for 1 h. The solution was cooled in an ice bath, acidified with HCl and filtered, yielding compound A (4.1 mmol; m.p. 265 °C, dec.).

4-((3-Methylpiperidin-1-yl)sulfonyl)aniline and 3-((3-methylpiperidin-1-yl)sulfonyl)aniline (B and B’, respectively, Figure S2) were prepared as follows: 4- or 3-nitrobenzenesulfonyl chloride (4.5 mmol) was dissolved in CH_2Cl_2 and 3-methylpiperidine (4.5 mmol) was added. A solution of TEA (4.5 mmol) in CH_2Cl_2 was added dropwise and the reaction was stirred at r.t. for 1h. The mixture was washed with dilute HCl and with water and the organic layer was dried and evaporated in vacuo, yielding 3-methyl-1-((4-nitrophenyl)sulfonyl)piperidine (3.6 mmol; m.p. 169-117 °C) and 3-methyl-1-((3-nitrophenyl)sulfonyl)piperidine (3.1 mmol; m.p. 109-110 °C). The nitro derivatives (1.9 mmol) were reacted with Sn (8.4 mmol) in EtOH (2 ml) and HCl 37% (5 ml), under reflux for 45’. After cooling, NaOH 20% and 20 ml Et_2O were added and the mixture was filtered through celite and washed with Et_2O. The organic layer was recovered, anhydrified and evaporated in vacuo, yielding amines B (1.1 mmol; m.p. 165-166 °C) and B’ (1.3 mmol; m.p. 119 °C). The chiral amines were prepared using the same protocol, but starting from (*S*)-3-methylpiperidine obtained as follows: 200 mmol of (+)-tartaric acid were dissolved in 80 ml of water. Then, 200 mmol of (±)-3-methylpiperidine were added and

the solution was left to stand overnight. The precipitate was collected and recrystallised from brine twice. The salt was then hydrolysed with a 30% ammonia solution and extracted with diethyl ether, yielding 14.8 mmol of pure (*S*)-3-methylpiperidine (yield 7%, $[\alpha]_D$: +1.9 (C= 0.01 g/ml, $CHCl_3$)).

Acid A (0.1 mmol) was refluxed in 5 mL of $SOCl_2$ for 6 h, then the excess of $SOCl_2$ was removed in vacuum. The crude was dissolved in toluene (10 mL) and TEA (0.1 mmol) and reacted with an equimolar amount of B or B' by stirring at r.t. overnight. After evaporation of the solvent, the crude was extracted in CH_2Cl_2/water and purified by silica gel chromatography (ethylacetate/cyclohexane 3:2) yielding compounds 3 or 3'. Purity was confirmed above 95% by HPLC.

3 and (*S*)-**3**: 5-(2-hydroxyphenyl)-*N*-(4-((3-methylpiperidin-1-yl)sulfonyl)phenyl)-1*H*-pyrazole-3-carboxamide; yield 45%, m. p. 254-258 °C; ^{1}H-NMR (300 MHz, DMSO) δ = 0.83 (d, J=6,23, 3H) 1.12-2.24 (m, 7H), 3.44-3.47 (m, 2H), 6.87-8.10 (m, 8H), 8.71 (s , 1H), 10,49 (bs, 1H). ^{13}C-NMR (75 MHz, DMSO) δ = 18.2, 23.6, 29.6, 45.6, 52.2, 59.2, 104.8, 115.8, 118.8, 119.3, 126.9, 127.9, 128.9, 129.0, 142.4, 153.8. ESI-MS *m/z* = 439.57[M -H]. HPLC: gradient 0-3 min (60% AcCN in H_2O) 3-23 min (from 60 to 100% AcCN); rt 6.4 min.

3' and (*S*)-**3**': 5-(2-hydroxyphenyl)-*N*-(3-((3-methylpiperidin-1-yl)sulfonyl)phenyl)-1*H*-pyrazole-3-carboxamide; yield 41%, m. p. 242 °C dec.; ^{1}H-NMR (300 MHz, DMSO) δ = 0.82 (d, J=6,6, 3H) 1.15-2.49 (m, 7H), 3.45-3.49 (m, 2H,) 6.86-8.15 (m, 8H), 8.28 δ (s, 1H), 10.50 (broad singlet, 1H); ESI-MS m/z = 439.48[M -H]. HPLC: gradient 0-3 min (60% AcCN in H_2O) 3-23 min (from 60 to 100% AcCN); rt 6.7 min.

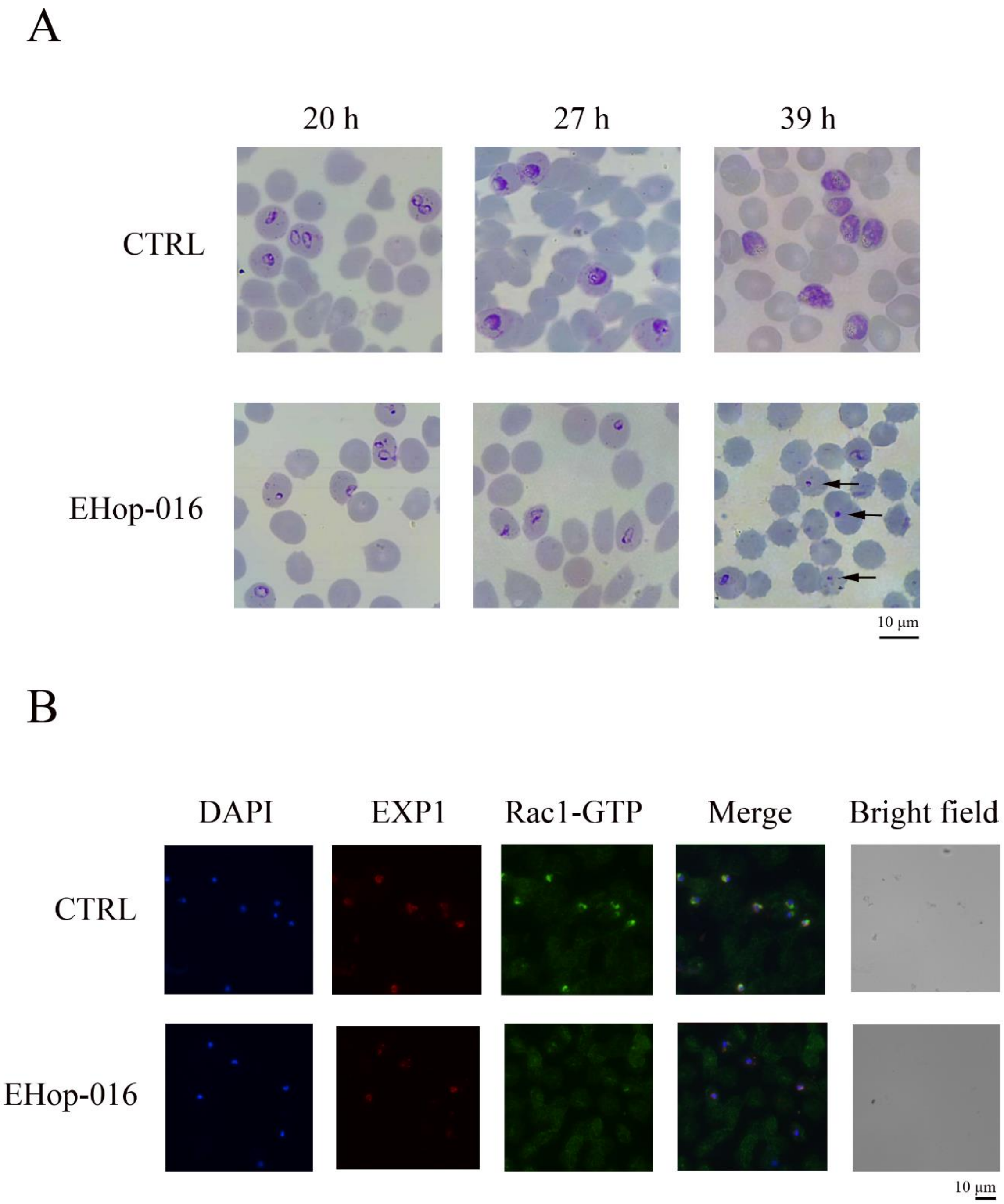

Figure S3. A. Representative images from Giemsa-stained smears from EHop-016-treated *P. falciparum* cultures at 20, 27 and 39 hpi and untreated control at the same time points. B. Immunofluorescence of synchronous infected erythrocytes at 20 hours post infection, treated

with 2.5 µM EHop-016 for 20 hours and untreated control, both stained with anti-Rac1/GTP antibody. BF: Bright field. Nuclei are stained with DAPI.

Table S1. Activity of pure enantiomers (S)-3 and (S)-3' against P. falciparum asexual parasites and gametocytes.

	**D10**<br>**IC_{50} (µM)**	**W2**<br>**IC_{50} (µM)**
**(*S*)-3**	0.58 ± 0.05	0.48 ± 0.03
**(*S*)-3'**	2.75 ± 0.27	1.13 ± 0.25

Table S2. Activity of CDC42 inhibitors against P. falciparum asexual parasites and gametocytes

	**D10**<br>**IC_{50} (µM)**	**W2**<br>**IC_{50} (µM)**	**3D7 mature gametocytes**<br>**IC_{50} (nM)**
**ZCL278**	>50.00	>50.00	63.48 ± 27.27
**MLS573151**	31.87 ± 6.54	39.69 ± 10.88	74.00 ± 19.59

References

1. D'Alessandro S, Corbett Y, Ilboudo DP et al. Salinomycin and other ionophores as a new class of antimalarial drugs with transmission-blocking activity. *Antimicrob Agents Chemother* 2015; **59**: 5135-44.
2. Lambros C, Vanderberg JP. Synchronization of Plasmodium falciparum erythrocytic stages in culture. *J Parasitol* 1979; **65**: 418-20.
3. Tribensky A, Graf AW, Diehl M et al. Trafficking of PfExp1 to the parasitophorous vacuolar membrane of Plasmodium falciparum is independent of protein folding and the PTEX translocon. *Cell Microbiol* 2017; **19(5):** e12710.
4. Barbey S, Goossens L, Taverne T et al. Synthesis and activity of a new methoxytetrahydropyran derivative as dual cyclooxygenase-2/5-lipoxygenase inhibitor. *Bioorg Med Chem Lett* 2002; **12**: 779-82.

5. Zagni C, Citarella A, Oussama M et al. Hydroxamic Acid-Based Histone Deacetylase (HDAC) Inhibitors Bearing a Pyrazole Scaffold and a Cinnamoyl Linker. *Int J Mol Sci* 2019; **20**.
